# Collaborating network in managing post the Mount Merapi’s disruption, Indonesia

**DOI:** 10.4102/jamba.v12i1.927

**Published:** 2020-09-15

**Authors:** Prawira Yudha Pratama, Achmad Nurmandi

**Affiliations:** 1 Department of Government Affairs and Administration, Universitas Muhammadiyah Yogyakarta, Yogyakarta, Indonesia

**Keywords:** collaborative governance, disaster management, social capital, PLS–SEM, stakeholders

## Abstract

Collaborative governance and social capital can help to form a resilient community in the wake of a disaster, such as the eruptions of Mount Merapi in Indonesia. This study examines the successfulness of the handling of disasters in Indonesia, with particular focus on eruptions of Mount Merapi. Disasters foster a close relationship between the government and the community in response to the emergency. This study uses a mixed method to analyse social networks in evaluating the structure of disaster networks in Indonesia and their implications for disaster management. Data were collected via a survey of 100 respondents from 28 institutions representing, for practical purposes, each population identified by each institution (government, non-governmental organisations and volunteers) that participated in handling the Merapi eruption disaster. The findings revealed that considerable miscommunication between institutions reduced the effectiveness of disaster management so that close discussion about conflict resolution was needed to develop more mature and systematic planning. Inter-agency trust is also felt to be necessary in disaster management. Trust between agency members and other institutions strongly supports the success of systematic disaster management. Meanwhile, every institution must foster open leadership by giving individuals with precise knowledge of the situation and the condition of the disaster area a mandate to lead directly in the field. Disaster governance is carried out through the agreement of each institution formed in the Disaster Emergency Planning (Rencana Penanggulangan Kedaruratan Bencana – RPKB) guidelines. These guidelines expect the Government and the community to coordinate with each other in a structured and systematic manner in the process of disaster management.

## Introduction

Indonesia, as an archipelago located on the equator, is prone to natural disasters. According to the National Disaster Management Agency (Badan Nasional Penanggulangan Bencana – BNPB), until mid-2018 a total of 1134 natural disasters had been recorded in Indonesia. These included tornadoes, landslides, floods, earthquakes and volcanic eruptions. Volcanic eruptions are frequent in Indonesia. This is not surprising because Indonesia has a record of 129 active volcanoes, 70 of which are considered very dangerous for the surrounding community (BNPB 2010).

Mount Merapi is a stratotype volcano with a height of 2980 m above sea level. Geographically, it is located at a latitude of 7°32.5′ South and a longitude of 110°26.5′ East. Administratively, it is located in the Sleman Regency in the Special Region of Yogyakarta and Magelang Regency, Boyolali Regency and Klaten Regency in Central Java Province. Mount Merapi has erupted numerous times. According to data from BNPB (2019), in the past few years, Mount Merapi has erupted approximately 40 times, with seven of these eruptions being significant. From 1768 to 1872, Mount Merapi has erupted more than 80 times, with major eruptions occurring in 1768, 1822, 1849 and 1872. Eruptions in this century have been more violent than were those in the 20th century, with hot clouds of ejecta reaching 20 km high. In the 20th century, there were at least 28 eruptions of Mount Merapi, with the most massive one occurring in 1931. There were fatalities in this incident; the damage increased to 13 villages, and 23 other communities were highly damaged. In 1933–1935 and 1961, eruptions caused lava flows. After that, in 1994, Mount Merapi ejected by breaking down the lava dome with a volume of 2.6 million m^3^. In that eruption, Mount Merapi spewed hot clouds of ejecta more than 6.5 km to the northwest and south, affecting 64 people badly and causing injuries. An eruption in 2006 destroyed the Kaliadem area, with two volunteers being killed by the hot cloud of the eruption. This eruption was marked by earthquakes and deformation of the landscape, followed by a rain of volcanic ash for 3 days in Magelang and Sleman, Central Java. The eruption of Mount Merapi in 2010 was the largest in the past 100 years. In mid-2018, Merapi began to exhibit increased activity, which continued until the end of January 2019. Merapi’s activity is still being monitored to minimise the danger it poses. Disasters require decentralised decision-making and intensive human interaction (Kapucu & Van Wart 2006; Kirschenbaum 2004). Managing disasters involves dynamic processes that are ideal yet demanding. Thus, collaboration between organisations and government agencies is essential for the development of an effective strategy and better performance during disasters.

Aside from the fact that collaboration often occurs between proximal and like agencies (Simo & Bies 2019), collaborative disaster management faces various challenges, which often leads to failure of the response operations. Poor communication, inadequate planning, misguided and poorly executed leadership and insufficient coordination with various stakeholders lead to collaborative failures (Streib & Waugh 2019; Wise 2019). Jatmiko and Tandiarrang (2014), in their study of the Indonesian Maritime Agency, found that the existing structure of the agency does not support good communication with agencies that are crucial to the agency’s performance. Meanwhile, Seng (2010) argued that the polycentric structure of Indonesian disaster management is ideal in responding to occurrences of tsunamis in the country. However, it does not fit with the norms of the Indonesian political community. Moreover, Nurmandi et al. (2015) studied different disasters in Indonesia and concluded that different structures are formed in the governance of each of the disasters they studied.

Emergency responses in Indonesia are often erratic, especially in terms of searching for and rescuing people and in coordinating both the collection and the distribution of aid to victims. Post-disaster recovery efforts are also not maximised. The inaccuracy of data in terms of the numbers of injured and the types of injuries suffered by victims makes it challenging to allocate the medical facilities needed for efforts to restore the health of victims. The complexity of the problem requires proper regulation, and careful planning of the response to disasters is needed to ensure that the implementation of disaster management will be more focused and integrated. Given the above problems, the Indonesian government’s implementation of regulations related to disaster management has not been optimal. This means that countermeasures that are taken do not follow systematic and planned steps. This study investigates the effect of cross-sector collaboration in disaster management by investigating the way in which collaborative governance is carried out by the government and non-governmental organisations in managing the Mount Merapi Eruption disaster in the Special Region of Yogyakarta.

## Literature overview: Collaborative governance in disaster management

For public sector organisations, the entire network approach is used widely as an analytical framework because it examines the connections that either exist or do not exist in a defined set of organisations, and this shows the extent to which organisations work with one another to achieve common goals (Provan & Kenis 2008).

Provan and Kenis (2008) examined multilateral relations that define the entire network and that are essential for achieving collective results. They suggested that the entire network must be analysed based on network governance, network leadership and management, and network performance. According to them, network governance refers to a network of coordination mechanisms and focuses on the network as a unit of analysis to guide the network in a steady state. There are three modes of network governance. The first mode is, shared or independent governance, which is characterised by easy form and high-level commitment but involves frequent meetings, lacks clear goals and experiences difficulties in reaching consensus. The second mode is leading the organisation, which refers to the efficiency of direction and precise network management but faces the potential for leader organisation dominance and low participation from members. The third mode is network administration organisations, which are entities that manage the networks but come with higher operating costs, more complex administrative processes, and the potential loss of control and decision-making authority for some network members. Meanwhile, Jung, Mazmanian and Tang (2009) suggested that leadership and management follow a framework that guides leaders and network managers between organisations in networks, irrespective of their chosen governance model.

Bryson, Crosby and Stone (2006) postulated that the structure of collaborative action changes and tends to be flexible because of membership ambiguity and complexity of the local environment. Such ambiguity arises from membership features, including perceptions about who is involved in the collaboration and what these members represent. Also, collaboration hierarchies, where individuals and organisations often become members of overlapping partnerships, further exacerbate the ambiguity of membership. Instead, governance between networks determines the survival and success of networks and collaboration. Bryson et al. (2006) viewed governance, characterised by initial agreements, leadership, planning, trust and conflict management, as a set of coordination and monitoring activities necessary for the network to survive. Governance is highly dependent on network structure, and as Bryson et al. (2006) emphasised, the choice of the type of governance structure tends to influence network effectiveness. The findings of Bryson, Crosby and Stone (2015) suggest that agreement is reached if public managers adopt an inclusive process that is made possible by flat structures.

Conversely, Dalton et al. (1980) explained that flat and tall structures pertain to the number of hierarchical levels of the organisation, where the span of control for the tall structure is narrower, whereas the span of control for the flat structure can be more comprehensive. They concluded that the organisation is structured in such a way that it fits its intended functions. And, therefore, the structure of the organisation may vary, but it may remain within a reasonable range in which there will be no difference in performance attributable to structure (Dalton et al. 1980).

### Previous performance

Primarily, government solutions in the form of policies and regulations are products of either market or sector failure. Lessons from past performance may suggest that sector failure serves as the foundation for committed sponsors and active champions to emerge and increase the informal and formal agreements in collaboration, particularly in terms of composition and accountability (Bryson et al. 2015). Moreover, trust, in terms of common bonds and confidence in organisation competence, might be developed despite failed efforts towards successful collaboration and can be a basis for starting new collaborative efforts (Rashid & Edmondson 2011). Meanwhile, the causes and implications of sector failure may lead to effective measures for managing conflict (Olu & Adesubomi 2014) and an effective planning process (Sial et al. 2013). Therefore, the previous performance of the network, which in this study refers to the Regional Disaster Management of the Special Region Yogyakarta, in terms of the targets and the performance indicators established in the Regional Disaster Management Plan (indicators for collaborative effectiveness), may have led to the existing relationship between network members (Quik et al. 2015). Hence, Hypotheses 1–6 are formulated:

H1: There is a significant relationship between previous performance and initial agreement.

H2: There is a significant relationship between previous performance and trust.

H3: There is a significant relationship between previous performance and planning.

H4: There is a significant relationship between previous performance and leadership.

H5: There is a significant relationship between previous performance and managing conflict.

H6: There is a significant relationship between previous performance and existing relationship.

### Initial agreement

Additionally, forging initial agreements by providing incentives and proper motivation mechanisms fosters inter-organisational communication and trust, improving inter-organisational network coordination in emergency management response operations (Ansell & Gash 2008; Kapucu 2006; Tang & Shui-Yan 2014). The studies of Jung et al. (2009) and Tang and Shui-Yan (2014) in the field of collaborative management discussed the importance of the right incentives as motivation and the various dynamics that went with them. Jung et al. suggested that the collaboration can be analysed by looking into how agencies and organisations perceive collaboration, their intentions in collaborating and their willingness to collaborate.

On the other hand, incentives and other motivation schemes should be implemented in the right sequence and manner to be effective (Tang & Shui-Yan 2014). Emerson, Nabatchi and Balogh (2011) explained public service motivation as ‘a general altruistic motivation to serve the interests of a community of people’, and self-sacrifice, as an aspect of public service motivation, makes an individual commit to organisational change (Wright et al. 2014). Therefore, providing the right kind of motivation demands a careful understanding of what the organisation needs and what inspires its members. As providing appropriate motivation mechanisms can lead to better performance, this study considers the initial agreement (Hypotheses 7–11) and measures it in the context of the motivation of the stakeholders and implementers in terms of altruism, organisational goals and increasing the legitimacy of the organisation (Andrews & Entwistle 2010):

H7: There is a significant relationship between initial agreement and leadership.

H8: There is a significant relationship between initial agreement and trust.

H9: There is a significant relationship between initial agreement and planning.

H10: There is a significant relationship between initial agreement and managing conflict.

H11: There is a significant relationship between initial agreement and existing relationship.

### Leadership

Moreover, managers need to understand and work strategically within the institutional environment and build capacity across agency boundaries through rigorous structures and processes with the new commitment and coordination required to work towards successful cross-agency collaborative management and a good network outcome (Kapucu 2006). Crucial to the success of the collaboration is the operationalisation of each member’s responsibility to continue the work and accomplish the goal despite the absence of leadership. Lester and Krejci (2019) explained that during disasters, the person exercising leadership is more important than is the person who is authorised to lead. On the other hand, poor communication, misguided and poorly executed leadership, lack of contingency plans and insufficient coordination with various stakeholders, in addition to insufficient preparation among communities, lead to either collaborative failures (Wise 2019) or sector failure (Bryson et al. 2006). Thus, Hypotheses 12–15 are made. This study measures leadership using six indicators with a 5-point scale. The indicators are vision, self-leadership, motivating and inspiring others, empowering people, collaborating and influencing and creativity and innovation (https://www.stepintoleadership.info/about.html):

H12: There is a significant relationship between leadership and trust.

H13: There is a significant relationship between leadership and planning.

H14: There is a significant relationship between leadership and managing conflict.

H15: There is a significant relationship between leadership and existing relationships.

### Trust

Significantly, Kapucu (2006) noted that effective response and recovery operations require collaborations and trust between government agencies at all levels and also between the public and nonprofit sectors. Generally, trust refers to a person’s confidence in the reliability of another person to produce specific outcomes, and the shared confidence held by the members of an organisation is called inter-organisational trust (Rashid & Edmondson 2011). The interdependencies among agencies and organisations through interactive processes such as face-to-face dialogues increase trust, build social capital and can develop into collaborative culture, which substantially increases the speed of decision-making and leads to successful collaborations (Ansell & Gash 2008; Emerson et al. 2011; Jung et al. 2009; Kapucu & Arslan 2015). Thus, this study considers trust, which is measured in terms of integrity, competence and dependability (Andrews & Entwistle 2010), as one of the aspects of governance. In doing so, Hypotheses 16–18 are tested:

H16: There is a significant relationship between trust and planning.

H17: There is a significant relationship between trust and managing conflict.

H18: There is a significant relationship between trust and existing relationships.

### Managing conflict

Meanwhile, the division of labour and different functions of the organisations in the network influence the attitude and behaviour of the members and inevitably create conflict. Thus, the interpersonal skills of the members and their relationship to the organisational integration should be explored (Lawrence & Lorsch 1967). Moreover, Bryson et al. (2006) elucidated the factors that influence the sustainability of the collaboration process: type of collaboration, power imbalances among members and competing for institutional logics within the collaboration. Vangen and Huxham (2019) believe that the power imbalances amongst collaborating partners cause mistrust and that this tends to worsen in cases of difficulty amongst partners in agreeing on a shared purpose. Hence, power imbalances and competing for institutional logics are a threat to effective collaboration, but with tactics such as strategic planning and scenario development, collaborations will likely succeed (Bryson et al. 2006). Thus, managing conflict, which is measured using the indicators related to team focus, personal style and action orientation (Managing Conflict at Work, BPBD), is considered in this study (Hypotheses 19 and 20):

H19: There is a significant relationship between managing conflict and planning.

H20: There is a significant relationship between managing conflict and existing relationships.

### Planning

**FIGURE 1:**
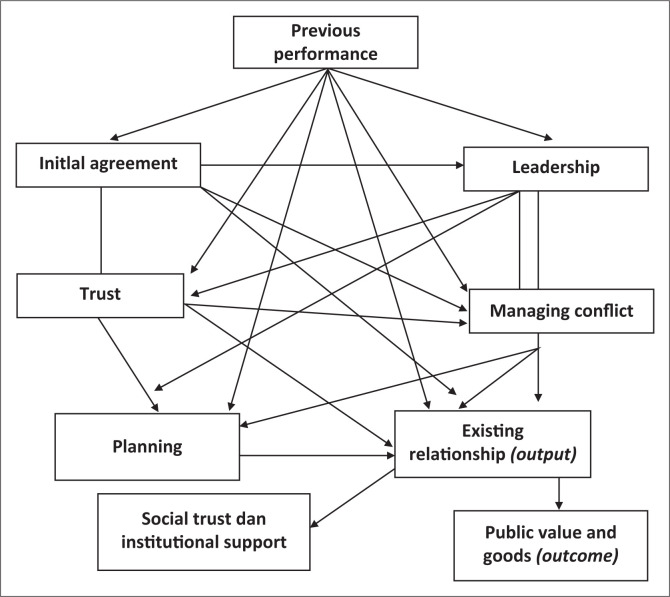
Mind map.

Lastly, an effective and emergent plan facilitates disaster management. It is argued that the leaders of the institutions involved in the disaster operations should participate in the planning process; otherwise, these leaders will attempt to assert themselves into the disaster situation despite earlier agreements put in place by either subordinates or predecessors, disrupting all disaster plans (Almarez et al. 2015; Lester & Krejci 2019). The disaster response operations during the World Trade Center attack, Hurricane Andrew and Hurricane Katrina revealed significant challenges, including either weak or non-existent planning, incompetent managers, political inattention before the event and political squabbling afterwards (Kapucu & Van Wart 2006). Thus, contingency plans can lead to fruitful collaborative disaster management (Hypothesis 21) (see Figure 1). As used in this study, a contingency plan is measured using the following indicators: conduct comprehensive needs’ assessment, determine objectives and strategies, and plan implementation and evaluation:

H21: There is a significant relationship between planning and existing relationships.

## Research methods

This research uses a mixed method. The combination method combines two types of research methods: quantitative analysis and qualitative research. In combined quantitative and qualitative research, the two methods are not applied simultaneously, but they can be applied interchangeably. Hence, in this study, the quantitative analysis uses research instruments, and the qualitative analysis is a descriptive research done to obtain a detailed picture of post-disruption disaster management. The analysis performed is regression analysis using structural equation modelling through partial least squares (PLS–SEM); this is used to test the relationship between variables. The study involved 100 respondents from 28 institutions, practically representing the entire population identified for this study. Data validity depends not only on the number and characteristics of respondents but also onthe type of questions asked. When the questions for this study were tested, the results revealed that the number of statement items contained 50 of 7 variables (initial agreement, trust, previous performance, managing conflict, leadership, planning and existing relationship) and the value of Cronbach’s alpha for the analysis was 0.912. This value implies that the questionnaire used in gathering primary data is valid and reliable.

## Findings

Analysis of the outer model ensures that the model with its variables and indicators is worth measuring, considering that this is valid and reliable. Thus, the external model analysis measures construct validity using convergent validity and discriminant validity as indicators.

As shown in Table 1, the highest heterotrait–monotrait ratio of correlations (HTMT) value among the variables is 0.772. Therefore, lower than the value of 0.85 or 0.90 is set. Also, the same findings apply to HTMT inference criteria that are defined by running a bootstrap routine. The bootstrap routine shows variable values below the confidence interval, and the confidence bias interval is corrected. All values differ significantly from 1. Therefore, discriminant validity is established for the outside models used in this study.

**TABLE 1:** Discriminant validity: heterotrait–monotrait ratio of correlations.

Variable	Existing relationship	Initial agreement	Leadership	Managing conflict	Performance	Planning	Trust
Existing relationship	0.729	-	-	-	-	-	-
Initial agreement	0.645	0.554	-	-	-	-	-
Leadership	0.535	0.730	0.761	-	-	-	-
Managing conflict	0.628	0.692	0.708	0.768	-	-	-
Performance	0.843	0.418	0.335	0.417	0.633	-	-
Planning	0.742	0.792	0.811	0.790	0.557	0.831	-
Trust	0.791	0.647	0.671	0.617	0.763	0.791	0.772

As revealed in Table 2, the structural model has a low predictive accuracy based on the values in Table 3. Furthermore, the *R*^2^ value of the existing relationship initial agreement and managing conflict is considered unacceptable. And that indicates that the independent variable (previous performance) cannot explain the endogenous dependent variable. However, *R*^2^ values are usually low in studies related to human behaviour and human relations with other humans because human behaviour is more difficult to predict (Sorrels et al. 2018). Therefore, even though the predictive capacity of structural models is low, important conclusions about the relationship between variables can still be derived from statistically significant predictors. As Paul (2017) said, a regression model with a low *R*^2^ value can be very good, for several reasons. Therefore, (high or low) *R*^2^ is not enough by itself.

**TABLE 2:** Hypotheses testing: Summary of the path analysis evaluation.

Hypothesised relationships	Original sample (O)	Sample man (M)	Standard deviation (STDEV)	*t-*statistics (O/STDEV)	*p*-values	Assessment of hypothesis
Initial agreement-existing relationship	0.182	0.176	0.160	1.190	0.335	Rejected
Initial agreement-leadership	0.715	0.726	0.095	7.523	0.000	Accepted
Initial agreement-managing conflict	0.192	0.186	0.190	1.190	0.235	Rejected
Initial agreement-planning	0.162	0.156	0.170	1.185	0.453	Rejected
Initial agreement-trust	0.174	0.176	0.176	1.190	0.235	Rejected
Leadership-existing relationship	0.152	0.176	0.180	1.160	0.635	Rejected
Leadership-managing conflict	0.162	0.126	0.160	1.130	0.244	Rejected
Leadership-planning	0.182	0.156	0.180	1.190	0.979	Rejected
Leadership-trust	0.360	0.235	0.181	2.812	0.004	Accepted
Managing conflict-existing relationship	0.182	0.166	0.170	1.190	0.256	Rejected
Managing conflict-planning	0.257	0.263	0.091	2.824	0.005	Accepted
Previous performance-existing relationship	0.134	0.166	0.190	1.130	0.756	Rejected
Previous performance-initial agreement	0.418	0.455	0.109	3.818	0.000	Accepted
Previous performance-leadership	0.843	0.843	0.028	3.518	0.000	Accepted
Previous performance-managing conflict	0.843	0.579	0.109	3.516	0.000	Accepted
Previous performance-planning	0.152	0.166	0.170	1.190	0.521	Rejected
Previous performance-trust	0.191	0.186	0.160	1.190	0.235	Rejected
Planning-existing relationship	0.182	0.176	0.198	1.180	0.296	Rejected
Trust-existing relationship	0.182	0.166	0.170	1.190	0.256	Rejected
Trust-managing conflict	0.192	0.176	0.180	1.196	0.215	Rejected
Trust-planning	0.843	0.843	0.028	30.191	0.000	Accepted

*Source*: Primary data processed by researchers

STDEV, standard deviation.

**TABLE 3:** Evaluation of structural models.

Variable	*R*^2^	Predictive Relevance (Q2)
Existing relationship	0.838	0.123
Initial agreement	0.175	0.068
Leadership	0.534	0.257
Managing conflict	0.584	0.023
Planning	0.840	0.273
Trust	0.784	0.324

Meanwhile, the effect size (f2) of the variables is also used to assess the structural model. The effect size (f2) values of 0.02, 0.15 and 0.35 for the significant independent variables represent weak, moderate and substantial effects, respectively (Chin 1998). The effect size of the hypothesised relationship between variables in this study, along with the *p*-value of the regression analysis.

### Hypothesis test: Regression analysis (structural equation modelling through partial least squares)

**FIGURE 2:**
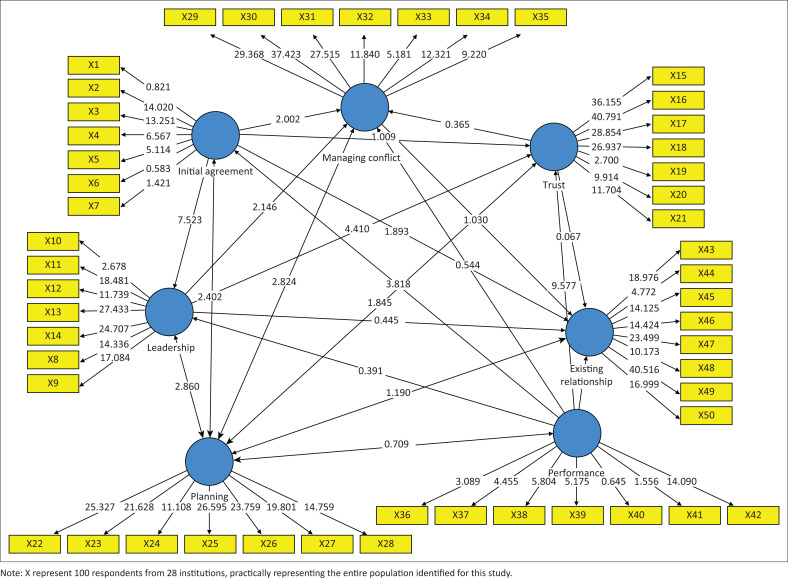
Structural model shows the corresponding Model P.

The hypothesis testing between variables is exogenous variable to endogenous variables; it is performed using a bootstrap resampling method after knowing the validity and reliability of the data. The statistical test used is the *t*-statistic or the *t*-test. Testing can be declared significant if the *t*-statistic value is > 1.96 and the *p*-values are < 0.05 (Haryono 2017). Hypothesis testing is done through knowing the output path coefficient of the bootstrap resampling results, which can be seen in the following table:

Figure 2 and Table 3 show the variables that do not have a significant influence on the latent variable. These 14 hypotheses have a *t*-statistic value that is lower than the criterion of > 1.96 (Table 2). The *p*-values of the five variables/hypotheses are higher than the *p*-value standard of < 0.05 (Haryono 2017).

### Hypotheses testing: Independent and dependent variables

#### Previous performance and initial agreement, leadership and managing conflict

Subsequently, the data show that previous performance has a significant relationship with the initial agreement of the network, which refers to altruism and the desire to increase the legitimacy of member institutions. As stated by the Head of the Office of the Regional Disaster Management Agency, ‘Members of the Provincial Disaster Risk Reduction and Management became more dynamic in their respective mandates, they participate actively in meetings, planning and decision-making process’. (Interview, 25 August 2019, Head of the Yogyakarta Special Region Disaster Management Agency, Mr. Biwara Yuswantana)

During an interview the Yogyakarta Special Region Social Service explained that:

‘As social workers, we work hard to collaborate more with the Subdistrict Health office and other related offices to be ready to respond more effectively during times of disaster. Managing conflicts also becomes accessible after the experience and performance of previous member institutions.’ (Mr Eko Suhargono, interview, 10 August 2019)

The disaster management division of the Indonesian Red Cross (PMI) explained that:

’As social workers, we work hard to collaborate more with the Subdistrict Health office and other related offices to be ready to respond more effectively during times of disaster. Managing conflicts also becomes accessible after the experience and performance of previous member institutions.’ (Mr. Haris Eko Yuliant, disaster management member, interview, 28 August 2019)

These data show that in general, the performance of previous disaster management networks significantly affected aspects of the governance process. Therefore, if the past network performance increases the motivation of members in institutions and leadership capacity and facilitates conflicts in the network, disaster management has a higher chance of being productive and successful.

#### Initial agreement and leadership

The initial agreement that referred to altruism and the desire to increase office legitimacy was significantly related to leadership. To quote from an interview from the Sleman Regency Regional Disaster Management Agency:

‘We, at our institution, understand the complexity and relevance of our mandate regarding disaster management. By doing that, we have created manuals and guidelines at all levels to be followed in the event of a disaster.’ (Mr. Joko Supriyanto, Disaster Management, interview, 09 August 2019)

As outlined by the Head of the Yogyakarta Special Region Disaster Management Agency, it was stated:

‘As the primary agency in disaster evacuation and camp management, amending the procurement process during the disaster is a great help for us in mobilising our resources to provide for the basic needs of the victims. We can now explore strategies that we can adopt to improve the delivery of our mandate. Therefore, in this case, the issuance of a new policy, namely the Policy Framework For Disaster Emergency Plan, or RPKB, can motivate member institutions to utilize their potential in fulfilling their respective mandates related to the disaster.’ (Mr. Biwara Yuswantana, interviewed, 25 August 2019)

As revealed, members of the disaster management network have a high level of altruism so that, despite challenges, they can explore ways to improve the delivery of their mandates. Therefore, altruistic tendencies of institutions and institutions to provide better public services, as well as their desire to increase each other’s legitimacy, contribute to better disaster leadership.

#### On leadership and trust

Table 2 also shows that leadership, which refers to creativity and innovation, collaboration, motivation and empowerment of people, has a statistically significant relationship with trust, which refers to competence and dependence. In general, leadership in disaster management networks must be effective in extracting the resources needed to ensure that members of the institution get what they need, especially during disasters. The PMI explained that:

‘Leadership in disaster management is well-defined but not always easy due to various issues that need to be addressed by the main institution, the government. Fortunately, leadership in disaster management can maintain the active participation of members over time, which assists us in becoming familiar with the mandates of other institutions that somehow easily help them whenever our agents have the means to do it.’ (Mr. Haris Eko Yulianto, disaster management member, interview, 28 August 2019)

There are several challenges in building an effective disaster response. The respondents stressed that the leadership of the Regional Disaster Management Agency could have facilitated various problems that occur during a disaster. For instance, there are general issues regarding the validity and reliability of information shared during disasters. Those issues cause doubts and miscommunication between agencies, which are discussed and solved in regular meetings before, during and after disasters to update and share true and real-time information with all stakeholders. The part of volunteers and community organisations articulates shares that:

‘Sharing information through regular government meetings, especially in times of disasters, or sending e-mails where possible and using handheld radios, help us in communicating the information needed to produce the right actions when we are given our responsibilities.’ (Volunteer, interview, 2019)

With this, competency as well as dependency between and amongst agents is increased. Meanwhile, the Indonesian Red Cross shares that:

‘We know that communication is very important in various aspects of our lives, but this cannot be managed perfectly during disasters when all lines of communication are cut off. Interruption in communication often arises; what we do is establish a command center where all messages are sent, and, most importantly, the feedback system is monitored to ensure that member agencies get accurate information.’ (Mr. Haris Eko Yulianto, disaster management member, interview, 28 August 2019)

#### On trust and planning

The strong relationship between trust and planning shows that each member shares the dependencies and competencies in institutions of the disaster management network, which leads to efficient network planning. According to the statement given by the Heads of each village affected:

‘We always believe that all member agencies are doing their best to fulfill their respective mandates, as we do. And when we are allowed to voice our concerns and thoughts during the meeting of all stakeholders in planning, we get to discuss and finally understand each other. As a result, I think we have made a better disaster management plan. Also, reliable exchange of information between agencies facilitates the planning activities of all the stakeholders included in the disaster network.’ (Mr. Suyatmi, interview, 25 August 2019)

Generally, the data show that the trust between members of disaster management networks in terms of competency and dependency among member institutions exceeds the complexity and challenges inherent in disaster management planning.

#### On managing conflict and planning

The significant relationship between managing conflict and planning reveals the importance of effective mechanisms in managing conflict towards more efficient disaster and emergency planning. As discussed before, conflict and disagreement cannot be avoided in any collaborative arrangement. Volunteers in the impacted area of the Merapi earthquake said:

‘During disasters, many individuals of their respective institutions want to be at the peak of response and rescue operations. However, when they face any bureaucratic procedures, they tend to take off and leave operations. As a result, misunderstandings arise between stakeholders.’ (Mr. Joko, volunteers and community organisations focal person, interview, 05 September 2019)

Disaster management always creates an atmosphere of misunderstanding between the government and non-government agencies. As stated:

‘Misconceptions in Disaster Management are usually caused by different interpretations of mandates or simple miscommunication between partner institutions. To solve them, discussions are held until all problems and problems are resolved. Occasionally, closed meetings are held to discuss and resolve conflicts.’ (Mr. Haris Eko Yulianto, disater management member, interview, 05 September 2019)

On the same note, representatives from community organisations described the conflict as being resolved professionally. The representatives from community organisations said to bear in mind that ‘whenever there is a difference, we discuss it among ourselves for a perfect solution. Usually, disagreements are discussed by groups such as shelter, health, water and sanitation’.

According to the volunteer representatives, everything was resolved properly, and it provided benefits in terms of decision-making and disaster management planning. As they said, ‘All stakeholders in disaster management ensure that conflicts are resolved before the actual meeting. As a result, consensus is reached’. Therefore, the network’s capacity to resolve conflicts because of coordination failure creates strong bonds and facilitates the disaster management network planning process.

## Discussion

The findings revealed that the previous performance of the network is crucially associated with the initial agreement, leadership and managing conflict, which validates the theory of Bryson et al. (2006). That sector failure facilitates cross-sector collaboration in terms of improving the initial agreement of the network as a way of making up for the shortcomings of particular sectors. The findings of Olu and Adesubomi (2014) are also supported as they stressed that a conflict management system based on the previous performance ensures a conducive environment in the process of collaboration. Lessons from sector failure serve as the basis for increasing informal and formal agreements in collaboration as well as enhancing the motivation mechanisms available (Bryson et al. 2015). Moreover, leadership in the network has a greater potential to evolve. Poor communication, misguided and poorly executed leadership by the federal and state government, and insufficient coordination with various stakeholders, as well as inadequate preparation among communities, lead to collaborative failures. And this study leads to the analysis of factors influencing the behaviour of public managers, such as the nature of the task performed and motivation mechanisms institutionalised in the network.

Moreover, the findings indicated that as leadership is significantly associated with the motivations in the initial agreement, leadership expands trust in the governance process. Generally, trust refers to a person’s confidence in the reliability of another person concerning certain outcomes, whilst the shared confidence held by the members of an organisation is called inter-organisational trust (Rashid & Edmondson 2011). The interdependencies amongst agencies and organisations through interactive processes such as face-to-face dialogues increase trust, build social capital and can develop into collaborative culture, which substantially increases the speed of decision-making and leads to fruitful collaborations (Ansell & Gash 2008; Emerson et al. 2011; Jung et al. 2009; Kapucu, Arslan & Demiroz 2010).

Significantly, the data show that trust in terms of dependability and competence is associated substantially with collaborative planning. The finding suggests that leadership enhances the reliability and expertise of the network, and trust enriches the planning process and output. These findings do not fully agree with the conclusions of Lester and Krejci (2019). Those findings postulated that the planning process should be participated by the leaders of the institutions involved in the disaster operations to ensure the successful result. However, this research supports the findings of Kapucu and Van Wart (2006) in the disaster response. Operations during the World Trade Centre attack, Hurricane Andrew and Katrina, when they postulated that the problems on weak or non-existent planning come along with incompetent managers. Thus, this research suggests that with good leadership, trust enhances the planning process of the network.

### Output and outcome of collaborative disaster

The outputs of collaborative governance show that cross-sector collaboration has a very direct influence on the management of Mount Merapi disaster, especially in the provision of mandates and information sharing. Meanwhile, the main problem during disaster management is the lack of available resources and the absence of reliable information. In addition, the less centralised nature of the disaster response network interferes with the governance process. The mandated network structure strengthens the capacity of key institutions to direct disaster response operations in the region. Kapucu (2006) asserts that the network must remain highly centralised in decision-making and decentralised in policy implementation. Data revealed that network members and their implementation shared decision-making.

## Conclusion

Given these findings, this study enriches the current understanding of cross-sector collaboration, which Bryson et al. (2006) referred to as an ideal but difficult and complicated approach towards the successful outcome. With its focus on the influences of the initial conditions to the aspects of governance process-leadership, initial agreement, trust, planning and managing conflict and its impact on the outcome of collaboration, this study reaffirms the previous studies conducted on cross-sector collaboration and disaster governance emphasising the relevance of the aspects of governance processes, particularly leadership in collaborative disaster management (Fung 2015; Jovita 2010; Kapucu et al. 2010; Lester & Krejci 2019). This study also joins the theoretical discussion on the relationship between the impact of the initial condition to the collaborative process that institutional design and sector failure, sets the basic ground under which collaboration takes place (Ansell & Gash 2008).

## Implications

This study further reinforces the findings of Chang Seng (2010) that a structure may be ideal, but that does not necessarily imply that it is suitable for every society because factors such as social norms and political culture may prove to be deterrents. This finding also confirms the study of Kapucu and Van Wart (2006), which shows that decentralised decision-making with excessive dependence on centralised authority can cause more harm than good, especially if the authority is not fully committed to addressing needs and solving various problems. There will be challenges along the way. This study enriches the existing understanding of cross-sector collaboration, which Bryson et al. (2006) referred to as an ideal but difficult and complicated approach towards a successful outcome.

Practically, this research implies that at the national and regional levels, where many organisations are part of the network, centralised decision-making is needed and disaster operations must be decentralised (Kapucu 2005). However, shared governance must be fostered in local government units where the network has relatively few members and very dense relationships can be established (Gheblawi et al. 2020; Provan & Kenis 2008), more importantly, a disaster management ambassador, known in Indonesia as *Pejuang Sigap Bencana*, who is committed to serving the public. Such an ambassador should adapt to changing operating conditions and ensure that the heads of institutions attend collaborative activities (Rai & Khawas 2019). Also, tensions can be avoided in implementing sufficient skills.
